# Injury severity levels and associated factors among road traffic collision victims referred to emergency departments of selected public hospitals in Addis Ababa, Ethiopia: the study based on the Haddon matrix

**DOI:** 10.1186/s12873-018-0206-1

**Published:** 2019-01-03

**Authors:** Ararso Baru, Aklilu Azazh, Lemlem Beza

**Affiliations:** 1 College of Medicine and Health Sciences, Arbaminch University, Arbaminch, Ethiopia; 2 Department of Emergency Medicine, School of Medicine, College of Health Sciences, Addis Ababa University, Addis Ababa, Ethiopia

**Keywords:** Road traffic accident, Injury severity, Haddon matrix, Ethiopia

## Abstract

**Background:**

Globally, about 1.25 million people die annually from road trafficcollisions. Evidence from global safety report shows a decreasing trend of road traffic injury indeveloped countries while there is an increasing trend in many developing countriesincluding Ethiopia. This study is aimed at assessing factors affecting injury severity levels of road traffic collision victims referred to selected public hospitals in Addis Ababa based on the Haddon Matrix.

**Methods:**

Ahospital-based cross-sectional study designwas implemented to randomly select a total of 363 road traffic collision victims. The collected data was cleaned andentered into Epidata version 3.1 and exported to SPSS Version 21 for analysis. Bivariate and multivariate logisticregression models were used to examine the association between explanatory and outcome variables.

**Results:**

A total of 363 individual sustained road traffic injuries were included to the study. Theprevalence of severe injury among road traffic accident victims was 36.4%. The following variables were significantly associated with increased injury severity: motorbike rider or motorbike passenger without helmet, adjusted odds ratio (AOR) 4.7(95% CI: 1.04–21.09); driving under the influence of alcohol, crude odds ratio (COR) 2.64(95% CI;1.23–5.64); victim with multiple injuries, AOR 3.88(95% CI: 2.26–6.65); vehicle size, AOR 2.14(95% CI: 1.01–4.52); collision in dark lighting condition, AOR 1.93(95% CI: 1.01–3.65); collision in cross city/rural, AOR 1.95(95% CI: 1.18–3.24) and vehicle occupant travelling unrestrained on the back of a truck, AOR3.9 (95% CI: 1.18–12.080). On the other hand, victims extricated at the scene by health care professional, AOR 0.33(95% CI: 0.13–0.83); victims extricated at the scene by police AOR 0.47(95% CI: 0.24–0.94); strict traffic police control at the scene of the collision, AOR 0.49(95% CI: 0.27–0.88) were significantly associated with less severe injuries.

**Conclusions:**

Findings reported in this paper suggest the need forimmediate and pragmatic steps to be taken to curb the unnecessary loss of livesoccurring on the roads. In particular, there is urgent need to introduce road safety interventions.

## Background

Globally, about 1.25 million people die annually from road traffic accident. This means more than 3400 death claims on adaily basis as a result of road traffic accident [1]. In addition, about 20 to 50 million people sustain nonfatal injuries as a result of road traffic crashes [2, 3]. The problem is anticipated tobecome the fifth leading cause of death with the annual death toll reaching 2.4 million by the year 2030 owing to an increased motor vehicle ownership and use associated with economic growth in developing countries [3, 4]. Indeed, it results in 3% loss of the gross domestic product worldwide and up to 5% in low and middle-income countries [1].

Accident pattern observed in developed countries show adecrease in road traffic accident while injury trends are notably increasing in middle and low-income countries including Ethiopia [3]. This trend will go further with thenoticeable disparity between developed and developing countries [2, 3].

In 2015, the proportion of vehicle was 46.6 per 1000 people in Africa while 510.3 per 1000 people in Europe. However, the highest death rate from road traffic accident recorded in Africa when compared with Europe stands at26.6 per 100,000 population versus 9.3 respectively [5].

In Ethiopia, road traffic collission is one of the critical road transport problem [6]. According to a 2015 global road safety report, the total numbers of vehicles registered in 2011/2012 Ethiopia fiscal year were 478,244. However, the WHO estimated fatality rates were 25.3 per 100,000 populations. This rate was far greater than rate registered in developed countries [1].

Even though Ethiopia has numerous problems related to road traffic safety, the study on road traffic collision (RTC) in the country is limited. Only afew published studiesshow theburden of road traffic accident in the country [7–12]. To the best of investigators’ knowledge, there is no study conducted on factors affecting injury severity of RTC in Ethiopia. As a result,the causal relationship between injury severity of road traffic accident victims and potential risk factors in Ethiopia remains unknown. So this study is aimed at assessing factors affecting injury severity levels of RTC victims referred to selected public hospitals in Addis Ababa based on Haddon Matrix.

## Methods

### Study setting and period

This study was conducted from March 1 to May 10, 2017 in selected public hospitals in Addis Ababa. The selected hospitals were the only hospitals in Ethiopia that provided trauma care at thenational level. These public hospitals were Tikur Anbessa Specialized Teaching Hospital (TASTH), St. Paul Millennium Medical College and Hospital (SPMMCH) and All Africa Leprosy, Tuberculosis, Rehabilitation and Training Center (ALERT) Hospital.

### Study design

A hospital-basedcross-sectional study design wasconducted to determine injury severity levels and associated factors at selected public hospitals in Addis Ababa, Ethiopia.

### Source population

All patients attending the Emergency Department of the above mentioned public hospitals in Addis Ababa due to road traffic collision injuries during the study period were the source population.

### Inclusion criteria, exclusion criteria and study subject

Road traffic collisionvictims who were referred to Emergency departments of selected public hospitals in Addis Ababa during the study period, regardless of their injury severity level and consented to participate were included in the study. However, victims or the family of the victims (for those unconscious and/or under 18 years old) that failed to give consent were excluded from the study. In addition, road traffic injuries as a result of non-motorized vehicles like bicycles and carts were excluded from this study.

### Sample size and sampling procedure

Sample size (n) was determined using single population proportion formula with the following assumptions: Based on the study conducted atBugando Medical Centre in Northwestern Tanzania the prevalence of severe road traffic injury was 38.6% [13] .The level of confidence (α) was taken as 0.05 (Z α) = 1.96); the margin of error was taken as 0.05.Accordingly, 363 road traffic collision victims were included in this study. Inaddition, to select study subject, sampling frame was developed from triage entry point and each respondent was accessed based on sampling frame by simple random sampling technique.

### Data Collectiontechniques and instruments

A pre-tested, structured, interviewer-administered questionnaire was used to collect data from study subjects. The questionnaire was developed after reviewing a number of literature [14–17]. The questionnaire has both open and close-ended questions. The key factors that were associated with road traffic collisions severity were classified based on Haddon Matrix. Furthermore, medical records of the victims were reviewed to check for consistency between information obtained from the interview and information recorded on the patient’s chart. Additional information were collected from police and medical staff in a condition that needs further information about the collision. The data collectors were Nurses. They were recruited based on their competence and data collection experiences.

### Measurement

Kampala Trauma Score II (KTS II) wasapplied to measure injury severity scores. It was adopted from aprevious study [18]. KTS II was applied to this study because of its similar performance with injury severity score (ISS), Revised Trauma Score (RTS), and Trauma Score and Injury Severity Score (TRISS) method to classify injury severity level [19]. Apart from this, the KTS II is considered as apotential tool for triage in resource-constrained setting [19]. And also, KTS II is able to provide areliable measurement for injury severity classification in emergency setting [18]. Indeed, KTS has clinically significant ability to predict theneed for hospitalization and fatality in resource-constrained settings [20, 21]. See (Table 1) for description of KTSII.

**Table 1:** Description of Kampala Trauma Score II (KTS II)

Label	Description		Score
A	Age (in years)	5–55	1
		< 5 or > 55	0
B	Systolic Blood pressure on admission	More than 89 mmHg	2
		Between 89 and 50 mmHg	1
		Equal or below 49 mmHg	0
C	Respiratory rate on admission	0–29/min	2
		30+	1
		≤9/min	0
D	Neurological status	Alert	3
		Responds to verbal stimuli	2
		Responds to painful stimuli	1
		Unresponsive	0
E	Score for serious injuries	None	2
		One injury	1
		More than one injury	0
Total (A + B + C + D + E) = __________________________			

### Operational definitions

#### Severe injury

Any RTC related injury resulting in a Kampala trauma score II of 6 or less [18].

#### Not severe injury

Any injuries resulting in a KTSII of 9 to 10 were considered as mild while KTSII of 7 to 8 were considered as a moderate [18]. However, for the purpose of this study, mild and moderate injuries were categorized under not severe injury.

### Data entry, processing,and analysis

The data was checked for completeness and consistency. Then it was cleaned and coded. The collected data was entered into EpiData version 3.1 (EpiData Association, Odense, Denmark) and then exported to SPSS version 21.0(IBM Corp., Armonk, NY, USA) for further statistical analysis.

Descriptive statistics were used to summarize the data. Bivariate logistic regression was used to explore the association of each independent variable with the dependent variable. Initially, thecrude odds ratio (COR) for each independent variable was calculated at 95% confidence interval (CI). All variables with *P*-value of < 0.25 were considered for multivariate logistic regression to control the effect of other confounders. Lastly, the significance level was set at *P* < 0.05.

### Ethical clearance

Ethical clearance was obtained from Addis Ababa University Emergency Medicine Department Research Ethics Committee (REC). Letter of permission was granted from TASTH, ALERT and AaBET administration officials. Informed consent was obtained from all conscious victims prior to proceeding data collection from them. The information collected from each participant was kept confidentially.

## Results

### Socio-demographic characteristics of the respondents

This study found that about three fourth 278(76.6%) of those who sustained RTC were males. Age group 21 to 30 years were mainly affected by RTC; followed by age group 12 to 20 years, and they account for 141(38.8%) and 74(20.4%) respectively (Table 2).

**Table 2:** Description of socio-demographic characteristics of the respondents

Variable	Categories	Frequency (Percentage)	Injury severity level		*x* ^*2*^
			Severe	Not severe	
Sex	Male	278 (76.6)	105	173	0.314
	Female	85 (23.4)	27	58	
Age	12 to 20	74 (20.4)	33	41	0.490
	21 to 30	141 (38.8)	49	92	
	31 to 40	70 (19.3)	22	48	
	41 to 50	48 (13.2)	16	32	
	> 50	30 (8.3)	12	18	
Occupation	Own work (including merchant)	136 (37.5)	45	91	0.738
	Driver	34 (9.4)	14	20	
	Government/Private employee	66 (18.2)	27	39	
	Student	54 (14.9)	20	34	
	Daily laborers	28 (7.7)	11	17	
	Farmers	31 (8.5)	12	19	
	Others^a^	14 (3.8)	5	9	
Region at which accident happened	Oromia	172 (47.4)	61	111	0.734
	Amhara	52 (14.3)	18	34	
	SNNPE	34 (9.4)	14	20	
	Addis Ababa	87 (24)	32	55	
	Others^b^	18 (4.9)	8	10	

^a^Driver assistant, retired, jobless

^b^Tigray, Benishangul, Harar, Afar, Gambella, Ethiopia Somali

### Basic characteristics of respondents

#### Host-related characteristics

About 144(39.7%) of the road traffic collision victims included in this study were pedestrians while 141(38.8%) of them were vehicle occupants. Concerning injury severity level, about 132(36.4%) of the road traffic collision victims sustained severe injuries while the rest of respondents sustained non-severe injuries (Table 3).

**Table 3:** Distribution of host-related characteristics

Variables	Categories	Frequency (Percentage)	Injury severity status		*x* ^*2*^
			Severe	Not severe	
Victims type	Pedestrian	144 (39.7)	52	92	0.081
	Driver	39 (10.7)	43	98	
	vehicle occupant	141 (38.8)	20	19	
	Motorbike rider or Occupant	39 (10.7)	17	22	
Duration of having driving license prior to accident^a^	≤2 years	107 (29.5)	43	68	0.474
	3 to 4 years	113 (31.1)	35	78	
	≥5 years	111 (30.6)	40	73	
Driver violate right of way	Yes	127 (35)	48	79	0.67
	No	236 (65)	84	152	
Driver used alcohol	Yes	34 (9.4)	19	15	0.011
	No	148 (40.8)	48	100	
	Unknown	182 (50.1)	66	116	
Multiple injuries	Yes	221 (60.9)	107	114	0.000
	No	142 (39.1)	25	117	
Driver used Seat belt (*N* = 39)	Yes	21 (53.8)	11	10	0.232
	No	18 (46.2)	6	12	
Vehicle occupant used Seat belt (*N* = 141)	Yes	17 (12.1)	6	11	0.261
	NO	124 (87.9)	42	75	
Motorist or occupant used helmet (*N* = 39)	Yes	17 (43.6)	5	12	0.016
	No	22 (56.4)	15	7	

^a^About 32 drivers either didn’t have driving license or unknown license status

#### Agent related characteristics

Majority 215(59.2%) of the RTC were happened by light vehicles followed by medium vehicles, 107(29.5%). In addition, collisions with pedestrian (144(39.7%) and vehicle to vehicle collisions71(27.3%) were the main collision types in this study respectively (Table 4).

**Table 4:** Distribution of vehicle and collission type

Variables	Categories	Frequency (Percentage)	Injury Severity status		x^2^
			severe	Not severe	
Vehicle type	Light vehicle	215 (59.2)	67	148	0.024
	Medium Heavy vehicle	107 (29.5)	44	63	
	Large Heavy Vehicle	41 (11.3)	21	20	
Accident type	Collision with pedestrian	144 (39.7)	52	92	0.045
	Collision with animate/an inanimate object	30 (8.3)	14	16	
	Vehicle to vehicle collision	71 (27.3)	16	55	
	Overturning	96 (26.4)	39	57	
	Falling from moving vehicle	22 (6.1)	11	11	

### Bivariate and multivariate analysis of factors associated with injury severity level

#### Host-related characteristics that determine road traffic collission severity level

In this study, victim type wasfound to have a statistically significant association with road traffic collission injury severity. Accordingly, vehicle occupants were 58 % less likely to be severely injured compared to pedestrians, AOR 0.42 (95% CI; 0.20–0.88) (Table 6).

A multivariate analysisshows that individual with multiple injuries was nearly four times more likely to have asevere injury than their counterparts, AOR 3.88(95% CI; 2.26–6.65) (Table 6).

Helmet utilization by motorist or motorbike occupants was associated with road traffic collission injury severity. Motorist or occupants who did not use helmet were nearly five times more likely to sustain a severe injury compared to those whoused a helmet (Table 6).

#### Agent related characteristics that determine road traffic collission severity level

Road traffic collision injury severity was associated with thetype of motor vehicle involved. This study depicted that victims involved in large heavy vehicle collission were 2.14 times more likely to develop severe injury than those involved in alight heavy vehicle with AOR 2.14(95% CI; 1.01–4.52) (Table 6).

Moreover, collissions occuringdue to two-vehicular crash were 52 % less likely to cause severe injuries compared to collissions occurring due tovehicle and pedestrian collisions after adjusting for potential confounders, AOR 0.48(95% CI; 0.24–0.93) (Table 6).

#### **Environmental characteristics that determine road traffic collissions** s**everity level**

Road traffic collissions which happened in dark environments were nearly two times more likely to be severe than those which happened in daylight with AOR 1.93(95% CI; 1.01–3.65). In addition, collissions which happened in across-city or rural area were 1.95 times more likely to be severe than road traffic collissions which happened in the urban area, AOR 1.95(95% CI; 1.18–3.24) (Table 6).

The accidents which happened to individuals in an environment with tight traffic police control were 51 % less likely to be severe injuries than aplace where there was no tight traffic police control, AOR 0.49(95% CI; 0.27–0.88). The availability of traffic signal or atoollike zebra crosswalk, traffic light, guardrail, pictures, symbols and speed breakers affects severity related to road traffic collissions. Collissions occurring in such environments were 42 % less likely to be severe than environments without them with AOR of 0.58(95% CI; 0.35–0.96) (Table 6).

Vehicle occupants seating location has astatistically significant association with road traffic collission injury severity in this study. Vehicle occupant travelling unrestrained on the back of a truck were nearly four times more likely to sustain severe injuries than vehicle occupants sat in the middle of apassenger vehicle, AOR 3.9(95% CI; 1.18–12.080) (Table 6).

Victims who were extricated at the scene by health care professionals were 67 % less likely to suffer severe injuries than those extricated by bystanders, AOR 0.33(95% CI; 0.13–0.83). Those extricated at the scene by police officers werefifty-3 % less likely to be severely injured than those extricated by bystanders with AOR of 0.47(95% CI; 0.24–0.94) (Table 6).

## Discussion

This study identified that the prevalence of severe injury among road traffic collission victims was 36.4%. This study’s finding was nearly similar to astudy conducted in Bugando Medical Center of Tanzania with 38.6% prevalence [13]. On the other hand, it was higher than the finding reported from Ethiopia and Kenya which were 10.87 and 19% respectively [7, 14]. The discrepancy could be due to the nature of the studies. This study was conducted in three public hospitals that mainly provide trauma care at the national level while the previous studiesin Ethiopia and Kenya were conducted inone hospital.

Regardingtheage of road traffic collision victims, majority 141(38.8%) of them were within the age group of 21–30 years (Table 2). This finding was in line with previous studies from Ethiopia [22, 23]. Concerning sex, males 278(76.6%) were more frequently affected by road traffic accident than females (23.4%). The higher male prevalence inroad traffic accidentswas previously reported by several studies [7, 13, 23, 24].

The proportion of RTCwas higher among pedestrians 144(39.7%) followed by vehicle occupants 141(38.8%) (Table 3). This finding was in agreement with previous studies conducted in Ethiopia and other studies from low and middle-income countries [8, 13]. This might be due to inadequate sidewalks for pedestrians, poor road design and inadequate road traffic signals in the country forpedestrians. It could be also due to inadequate public awareness of road traffic rules, thediscourteous behavior of drivers or motorists, violation of traffic rules by drivers and pedestrians in the country [23].

The Ethiopian government is enforcing preventive measuressuch as seat belt use for both drivers andvehicle occupants, and helmet use for both motorists and motor occupants [1]. However, only 17(12.1%) of the vehicle occupants and 21(53.8) of injured driver used seat beltswhile 17(43.6%) of the motorist or motorbike occupants used ahelmet (Table 3). The latter finding was similar witha studydone in Tanzania, 43.3% [24].

Majority of the collisions happened in the daylight, 260(71.6%) (Table 5). This finding was consistent with other studies [13, 23]. In addition, themajority of the collissions occurred in urban settings, 195(53.7%). This finding was in contrast to the study done in Iran [15]. The existence of traffic jam during the daytime, poor road network and mixed traffic flow system in urban areas might be the reasons forahigher collision during daylight and in urban areas [25].

**Table 5:** Environmental characteristics of RTC victims. Environment-related characteristics of respondents

Variables	Categories	Frequency (percentage)	Severity status		x2
			Severe	Not severe	
Time of collission	8 am to 2 pm	144 (39.7)	52	92	0.471
	2 pm to 8 pm	127 (35)	41	86	
	8 pm to 2 am	45 (12.4)	20	25	
	2 am to 8 am	47 (12.9)	19	28	
Lighting condition	Day light	260 (71.6)	88	172	0.039
	Dusk or dawn	40 (11)	13	27	
	Dark	63 (17.4)	31	32	
Place of collission	Urban road	195 (53.7)	55	140	0.000
	Rural/cross city road	168 (46.3)	77	91	
Weather condition	Raining	65 (17.9)	20	45	0.431
	Not raining	298 (82.1)	113	185	
Road surface condition	Asphalt	324 (89.3)	120	204	0.442
	Gravel	39 (10.7)	12	27	
Availability of Safety tools or signals	Yes	117 (32.2)	33	84	0.030
	No	230 (63.4)	92	138	
	Unknown	16 (4.4)	8	8	
Persons extricated the victim at the scene	Bystanders	266 (73.3)	107	159	0.039
	Police	64 (17.6)	17	47	
	Healthcare professionals	33 (9.1)	8	25	
Received pre hospital care	Yes	52 (14.3)	14	38	0.126
	No	311 (85.7)	118	193	
Tight traffic police monitoring	Yes	99 (27.3)	22	77	0.001
	No	264 (72.7)	110	154	
Mode of transport	Ambulance	89 (24.5)	31	58	0.865
	Other motorized Vehicle	252 (69.4)	92	160	
	Carried by people or non-motorized transportation	22 (6.1)	9	13	
Pedestrian location from the road at the moment of collission (*N* = 144)	Middle of the road	82 (56.9)	32	50	0.579
	Left side for pedestrian	30 (20.8)	9	21	
	right side for pedestrian	32 (22.2)	10	22	
Vehicle occupant seating location (*N* = 141)	Front seat of any vehicle	52 (36.9)	12	40	0.042
	Middle seat	54 (38.3)	14	40	
	Rear seat	16 (11.3)	6	10	
	At the back of truck	19 (13.5)	10	9	

Majority of the victims arrived healthcare facilities by private vehicles, 252(69.4%), followed by ambulances 89(24.5%) (Table 5). Though the proportion of victims that arrived the health facilities by ambulance was low, this finding is slightly higher than the result reported by previous studies in Addis Ababa [8, 22]. Concerning prehospital care, only 52(14.3%) of the victims had prehospital care. This finding washigher than reports from previous studies in Ethiopia and Tanzania, which reported 0 % prehospital services for RTA victims [7, 13]. The higher ambulance utilizations and the prehospital services received by victims in this study could be due to the establishment of organized prehospital services in Addis Ababa and involvement of private business groups inthe ambulance and the pre-hospital services such as Tebita Ambulance in Addis Ababa.

The drivers who drove under influence of alcohol were 2.64 times more likely to cause severe injury to themselves or to others than when compared with their counterparts on bivariate analysis, COR 2.64(95% CI; 1.23–5.64). However, it is statistically not significant on multivariate analysis, AOR 2.1(95% CI; 0.93–4.71) (Table 6). Alcohol consumption and driving had a clear effect on injury severity as reported by previous studies from Philippines, United States and Canada [26–28].

**Table 6:** Bivariate and multivariate analyses of factors affecting injury severity levels of road traffic collission victims

Variable	Categories	Injury severity level		COR 95% CI	AOR 95% CI
		Severe	Not severe		
Victims type	Pedestrian	52	92	1	
	Driver	17	22	1.36 (0.67–2.80)	1.11 (0.53–2.32)
	Motorist/Motor occupant	20	19	1.86 (0.91–3.80)	1.56 (0.74–3.26)
	Vehicle occupant	43	98	0.78 (0.47–1.27)	0.42 (0.20–0.88)^*^
Driver used alcohol	Yes	19	15	2.64 (1.23–5.64)^*^	2.1 (0.93–4.71)
	No	48	100	1	1
Motorist/motorbike occupant used helmet	Yes	5	12	1	1
	No	15	7	5.14 (1.30–20.36)	4.7 (1.04–21.09) ^**^
Presence of multiple injuries	Yes	107	114	4.4 (2.65–7.29)	3.88 (2.26–6.65) ^***^
	No	25	117	1	1
Vehicle type	light vehicle	67	148	1	1
	medium heavy vehicle	44	63	1.54 (0.95–2.50)	1.62 (0.96–2.75)
	large heavy vehicle	21	20	2.31 (1.18–4.56)	2.14 (1.01–4.52) ^*^
Crash type	Crash with Pedestrian	52	92	1	1
	Two vehicle collision	16	55	0.51 (0.27–0.99)	0.48 (0.24–0.93)^*^
	Over turning	38	57	1.18 (0.69–2.01)	1.38 (0.65–2.92)
	Animate/inanimate	14	16	1.55 (0.70–3.42)	1.34 (0.59–3.01)
	Falling from moving vehicle	12	11	1.93 (0.80–4.68)	1.45 (0.58–3.64)
Lighting Condition	Daylight	88	172	1	1
	Dusk or dawn	13	27	0.94 (0.46–1.91)	0.99 (0.45–2.17)
	Dark	31	32	1.89 (1.08–3.30)	1.93 (1.01–3.65) ^*^
Place of accident	Urban	55	140	1	1
	Cross city/rural	77	91	2.15 (1.39–3.33)	1.95 (1.18–3.24) ^**^
Traffic signals or safety tools available	Yes	32	85	0.59 (0.36–0.95)	0.58 (0.35–0.96) ^*^
	No	93	137	1	1
Persons extricating the victim from scene	Bystanders	107	159	1	1
	Police	17	47	0.54 (0.29–0.99)	0.47 (0.24–0.94) ^*^
	Healthcare professionals	8	25	0.48 (0.21–1.09)	0.33 (0.13–0.83) ^*^
Received pre-hospital care	Yes	14	38	1	
	No	118	193	1.66 (0.86–3.19)	1.23 (0.61–2.51)
Traffic police control at the scene	Yes	22	77	0.40 (0.23–0.68)	0.49 (0.27–0.88)^*^
	No	110	154	1	1
Vehicle occupant seating position	Front seat	12	40	0.86 (0.35–2.08)	1.21 (0.44–3.28)
	At the back of truck	10	9	3.17 (1.01–9.41)	3.9 (1.18–12.080)^*^
	Rear seat	6	10	1.71 (0.53–5.58)	1.95 (0.53–7.23)
	Middle seat	14	40	1	1

*P < 0.05, **P < 0.01, ***P < 0.001

The protective effect of helmet use on injury outcomes has been well documented in previous studies [29, 30]. In line with other studies, the present study found statistically significant association between injury severity level and helmet use on multivariate analysis, AOR 4.7(95% CI; 1.04–21.09) (Table 6).

The study revealed that vehicle to vehicle collisions were 52% less likely to cause severe injury than vehicle to pedestrian collisions, AOR 0.48(95% CI; 0.24–0.93) (Table 6). A study from Iran and Germany also reported existence of association between crash type and injury severity [15, 31]. Moreover, thecrash involved large heavy vehicles were 2.14 times more likely to be severe thanlight vehicles with AOR of 2.14(95% CI; 1.01–4.52). This finding is in agreement with other studies [32–35].

The collisions happening in dark conditions were almost two times more likely to be severe thanthose happening indaylight, AOR 1.93(95% CI; 1.01–3.65) (Table 6). This finding is consistent with other studies conducted in the developing and developed theworld [14, 17, 26, 27, 36].

A road traffic collission that occurred in thecross-city or rural environment is more likely to be severe than collissions that happened in urban areas, AOR 1.95 (95% CI; 1.18–3.24) (Table 6). This finding is consistent with the study conducted in Sweden [37]. This might be attributed to excessive speeding, low traffic police presence, inadequacy or absence of emergency medical services, and greater distance to hospitals in the rural areas [7].

Victims who sustained road traffic injury in environments equipped with safety tools liketraffic lights, guardrails, speed breakers and safety signals such as traffic symbols, pictures,and zebra crosswalk were 42% less likely to sustain severe injuries than their counterparts with AOR of 0.58(95% CI; 0.35–0.96). Furthermore, this study shows that injuries occurring in environments with tight traffic police control were 51% less likely to be severe than those occurring in locations without tight traffic police control, AOR 0.49(95% CI; 0.27–0.88) (Table 6). This finding was consistent with thestudy conducted in Bangladesh [17].

Victims extricated from collission scenes by health care providers and by the police were 67 and 53% less likely to sustain severe injury respectively than those extricated by ‘Good Samaritans’with AOR of 0.33(95% CI; 0.13–0.83) and 0.47(95% CI; 0.24–0.94) respectively (Table 6). This finding is in agreement with the study conducted in Iran [38].

### Limitations of the study

Self-reporting of certain variables may have caused overestimation or underestimation of the outcomes. This also may have caused possible bias in some individual responses from fear of legal punishment, which has a tendency to underestimate or overestimate the association. This study excluded vehicle speed at the moment of collission due to missing data and exaggerated response bias. Moreover, no restriction was placed on the vehicle model year in this study.

## Conclusion

This study found helmet use,victim type and presence of multiple injuries as the most important host-related factors that determine RTC injury severity levels. Meanwhile, vehicle type and crash type were agent related determinant of injury severity. In addition, lighting condition, place of collissions, the seating position of thevehicle occupant, availability of traffic signals and tools at accident location, availability of tight traffic police control and the persons who extricated the victim from the scene of collissions were among environmental factors that determine injury severity levels.

Results reported in this paper also suggest the need for immediate and pragmatic steps to be taken to curb the unnecessary loss of lives occurring on the roads. In particular, there is urgentneed to introduce road safety interventions that target basic identified factors in this study (host-agent and environment) and time sequence of collissions (pre-crash, crash and post-crash events).

## Abbreviations

AA: Aklilu Azazh
AaBET Hospital: Addis Ababa burn and trauma hospital
AB: Ararso Baru
AOR: Adjusted odds ratio
COR: Crude odds ratio
ISS: Injury severity score
KTS II: Kampala trauma score II
LB: Lemlem Beza
MAIS: Maximum abbreviated injury scale
REC: Research Ethics Committee
RTC: Road traffic collision
RTS: Revised trauma score
SNNPE: Southern nations and nationalities, and peoples of Ethiopia
SPMMCH: St. Paul Millennium Medical College and Hospital
SPSS: Statistical package for social Science
TASTH: Tikur Anbessa Specialized Teaching Hospital
TRISS: Trauma score and injury severity score

## References

[1] World Health Organization. Global Status Report on Road Safety 2015. WHO Libr Cat Data Glob [Internet]. 2015;340. Available from: http://www.who.int/violence_injury_prevention/road_safety_status/2015/en/.

[2] World Health Organization. Global status report on road safety: time for action. WHO Libr Cat Data.Geneva; 2009. Available from (www.who.int/violence_injury_prevention/road_safety_status/2009).

[3] World Health Organization. Global status report on road safety: supporting a decade of action. In: WHO Libr Cat Data Glob. Geneva; 2013. Available from http://www.worldcat.org/title/global-status-report-on-road-safety-2013-supporting-a-decade-of-action/oclc/889616028;

[4] World Health Organization. The global burden of disease: 2004 update. WHO Libr Cat Data Glob. 2008. Available from: https://www.who.int/healthinfo/global_burden_disease/GBD_report_2004update_full.pdf?ua=1;

[5] World Health Organization. Road safety in the African region. In: WHO Libr Cat Data Glob; 2016. Available from : https://www.who.int/violence_injury_prevention/road_safety_status/2015/en/;

[6] United Nations Economic Commission for Africa. Case Study: Road Safety in Ethiopia Final Report. 2009;(ECA/NRID/019). Available from: http://hdl.handle.net/10855/5250

[7] Seid M, Azazh A, Enquselassie F, Yisma E. Injury characteristics and outcome of road traffic accident among victims at Adult Emergency Department of Tikur Anbessa specialized hospital, Addis Ababa, Ethiopia: a prospective hospital based study. BMC Emerg Med. 2015;15:10. Published 2015 May 20. 10.1186/s12873-015-0035-4. Available from: https://www.ncbi.nlm.nih.gov/pmc/articles/PMC4493961/10.1186/s12873-015-0035-4 PMC4493961 25990560

[8] Getachew S, Ali E, Tayler-Smith K, et al. The burden of road traffic injuries in an emergency department in Addis Ababa, Ethiopia. Public Health Action. 2016;6(2):66–71. Available from: https://www.ncbi.nlm.nih.gov/pmc/articles/PMC4913687/10.5588/pha.15.0082 PMC4913687 27358798

[9] Mekonnen FH, Teshager S (2014). Road traffic accident: the neglected health problem in Amhara National Regional State, Ethiopia. Ethiop J Health Dev.

[10] Fekede A, Demeke A, Tesfaye G (2014). Magnitude of, trends in, and associated factors of road traffic collision in Central Ethiopia. BMC Public Health.

[11] Hailemichael F, Suleiman M, Pauolos W. Magnitude and outcomes of road traffic accidents at hospitals in Wolaita zone, SNNPR, Ethiopia. BMC Research Notes. 2015;8(135). Available from : https://bmcresnotes.biomedcentral.com/articles/10.1186/s13104-015-1094-z 10.1186/s13104-015-1094-z PMC4404595 25886357

[12] Woldeyohannes SM, Moges HG (2014). Trends and projections of vehicle crash related fatalities and injuries in Northwest Gondar. Ethiopia: A time series analysis Int J Env Health Eng.

[13] Chalya PL, Mabula JB, Dass RM, Mbelenge N, Ngayomela IH (2012). Injury characteristics and outcome of road traffic crash victims at Bugando medical Centre in Northwestern Tanzania. J Trauma Manag Outcomes [Internet].

[14] Mogaka EO, Ng’ang’a Z, Oundo J, Omolo J LE. Factors associated with severity of road traffic injuries, Thika, Kenya. Pan Afr Med J. 2011;8(20). Available from: http://www.panafrican-med-journal.com/content/article/8/20/full/10.4314/pamj.v8i1.71076 PMC3201586 22121429

[15] Masoumi K, Forouzan A, Barzegari H (2016). Effective factors in severity of traffic accident-related traumas; an epidemiologic study based on the Haddon matrix. Emergency.

[16] Tavakoli A, Shariat-mohaymany A, Ranjbari A (2012). Analysis of factors associated with traffic injury severity on rural roads in Iran. Journal of Injury and Violence Research.

[17] Kamruzzaman M, Haque MM, Ahmed BYT (2013). Analysis of traffic injury severity in a mega City of a developing country.

[18] Mutooro SM, Mutakooha EKP (2010). A comparison of Kampala trauma score II with the new injury severity score in Mbarara University teaching hospital in Uganda. East Cent African J Surg.

[19] MacLeod JB, Kobusingye O, Frost C, Lett R, Kirya FSC (2003). A Comparison of the Kampala Trauma Score ( KTS ) with the Revised Trauma Score ( RTS ), Injury Severity Score ( ISS ) and ... A Comparison of the Kampala Trauma Score ( KTS ) with the Revised Trauma Score ( RTS ), Injury Severity Score ( ISS ) and the TRI. Eur J Trauma.

[20] Gardner A, Kobina P, Oduro G, Stewart B, Dike N, Glover P, et al. Diagnostic accuracy of the Kampala Trauma Score using estimated Abbreviated Injury Scale scores and physician opinion. Injury [internet]. Elsevier Ltd; 2017;48(1):177–183. Available from: 10.1016/j.injury.2016.11.022 10.1016/j.injury.2016.11.022 PMC5203935 27908493

[21] Weeks SR, Juillard CJ, Monono ME, Etoundi GA, Ngamby MK, Hyder AASK (2014). Is the Kampala trauma score an effective predictor of mortality in low-resource settings? A comparison of multiple trauma severity scores. World J Surg.

[22] Tiruneh BT, Dachew BA, Bifftu BB. Incidence of Road Traffic Injury and Associated Factors among Patients Visiting the Emergency Department of Tikur Anbessa Specialized Teaching Hospital , Addis Ababa , Ethiopia Hindawi Publishing Corporation; 2014;2014:0–5. 10.1155/2014/439818 PMC4140128 25165583

[23] Asefa F, Assefa D, Tesfaye G (2014). Magnitude of , trends in , and associated factors of road traffic collision in central Ethiopia. BMC Public Health.

[24] Respicious Boniface, Lawrence Museru, Othman Kiloloma, Victoria Munthali. Factors associated with road traffic injuries in Tanzania. Pan Afr Med J [Internet]. 2016;8688:1–7. Available from: http://www.panafrican-med-journal.com/content/article/23/46/full/10.11604/pamj.2016.23.46.7487 PMC4862800 27217872

[25] Persson A. Road traffic accidents in Ethiopia : magnitude , causes and possible interventions. Adv Transp Stud an Int J. 2008;15(Section A):2008 Available from. http://www.atsinternationaljournal.com/index.php/2008-issues/xv-july-2008/414-road-traffic-accidents-in-ethiopia-magnitude-causes-and-possible-interventions

[26] Mao Y, Zhang J, Robbins G, Clarke K, Lam M, Pickett W (1997). Factors affecting the severity of motor vehicle traffic crashes involving young drivers in Ontario. Inj Prev [Internet].

[27] Rice TM (2003). Nighttime driving, passenger transport, and injury crash rates of young drivers. Inj Prev.

[28] Connor LRO, Andres R, Ruiz L (2014). Alcohol and hospitalized road traffic injuries in the Philippines. YALE J Biol Med.

[29] Chang F, Li M, Xu P, Zhou H, Haque M (2016). Injury Severity of Motorcycle Riders Involved in Traffic Crashes in Hunan , China: A Mixed Ordered Logit Approach. Int J Environ Res Public Health.

[30] Kim C, Wiznia DH, Averbukh L, Feng D, Leslie MP (2015). The economic impact of helmet use on motorcycle accidents: a systematic review and meta-analysis of the literature from the past 20 years. Traffic Inj Prev.

[31] Decker S, Otte D, Muller CW, Omar M, Krettek C, Brand S. Road Traffic Related Injury Severity in Truck Drivers : A Prospective Medical and Technical Analysis of 582 Truck Crashes. Arch Trauma Res. 2016;5(2). 10.5812/atr.31380 PMC5035669 27679790

[32] Pfortmueller CA, Marti M, Kunz M, Lindner G, Exadaktylos AK (2014). Injury severity and mortality of adult zebra crosswalk and non-zebra crosswalk road crossing accidents: a cross-sectional analysis. PLoS One.

[33] Cirera, E., Plasència, A., Ferrando, J., & Seguí-Gomez M. Factors associated with severity and hospital admission of motor-vehicle injury cases in a southern european urban area. Annu Proc / Assoc Adv Automot Med [Internet]. 1998;42:287–302. Available from: https://www.ncbi.nlm.nih.gov/pmc/articles/PMC3400211/pdf/aam42_p287.pdf 10.1023/a:1017961921607 11680536

[34] Tefft BC (2013). Impact speed and a pedestrian’s risk of severe injury or death. Accid Anal Prev.

[35] Roudsari B, Mock C, Kaufman R, Grossman D, Henary B, C.J. (2004). Pedestrian crashes: higher injury severity and mortality rate for light truck vehicles compared with passenger vehicles. Injury Prevention.

[36] Quddus MA, Noland RB, Chin HC (2002). An analysis of motorcycle injury and vehicle damage severity using ordered probit models. J Saf Res.

[37] Tainio M, Olkowicz D, Teresinski G, de Nazelle A, Nieuwenhuijsen MJ (2014). Severity of injuries in different modes of transport, expressed with disability-adjusted life years (DALYs). BMC Public Health.

[38] Khorasani-zavareh D, Khankeh HR, Mohammadi R, Laflamme L, Bikmoradi A, Haglund BJA (2009). Post-crash management of road traffic injury victims in Iran . Stakeholders â€™ views on current barriers and potential facilitators. BMC Emerg Med.

